# Real-Time Automated Fluorescence Imaging Assay for Quantifying GFP-Expressing *Mycobacterium avium* subsp. *paratuberculosis*

**DOI:** 10.3390/ijms27219640

**Published:** 2026-10-28

**Authors:** Gerard Badia-Bringué, Arrate Prado-López, Marta Alonso-Hearn

**Affiliations:** 1 Department of Animal Science, NEIKER-Basque Institute for Agricultural Research and Development, Basque Research and Technology Alliance (BRTA), 48160 Derio, Bizkaia, Spain; gbadia@neiker.eus (G.B.-B.); arrate.prado@neiker.eus (A.P.-L.); 2 Doctoral Program in Immunology, Microbiology, and Parasitology, Euskal Herriko Unibersitatea (EHU), 48940 Leioa, Bizkaia, Spain

**Keywords:** *Mycobacterium avium* subsp. *paratuberculosis*, real-time fluorescence microscopy, mycobacterial growth inhibition assay (MGIA), IncuCyte^®^

## Abstract

Measurement of *Mycobacterium avium* subsp. *paratuberculosis* (MAP) load within macrophages using a mycobacterial growth inhibition assay (MGIA) is an accurate indicator of host immune control of MAP infection. Real-time fluorescent microscopy allows the continuous analysis of infected cells and the quantification of fluorescence intensity, which directly correlates with concentration of fluorescent bacteria. The objective of this study was to develop an automated and real-time live-cell imaging assay using the IncuCyte SX1^®^ platform for quantifying green fluorescence protein (GFP)-expressing MAP (MAP-GFP) within infected macrophages. The fluorescence of serially diluted free MAP-GFP was quantified using the IncuCyte SX1^®^ demonstrating that the fluorescence signal of 3 × 10^7^ CFU was significantly different from lowest MAP concentrations. Significant differences between uninfected and infected BOMAC cells (MOI; 1:10) in DMEM-F12, DMEM-F12 supplemented with fetal bovine serum (FBS), and RPMI-1640 supplemented with FBS were observed, indicating the suitability of these culture media for measuring MAP-GFP-host cell interaction in the IncuCyte SX1^®^. For the optimization of the MOI and cell density, bovine monocyte-derived macrophages (MDMs) and the BOMAC cells were seeded at different cell densities and infected with MAP-GFP at different MOIs. After 2 h of infection, the supernatant was removed, the cells were washed three times with HBSS, and fresh RPMI-1640 supplemented with FBS was added to the cells. Then, the fluorescence was quantified in the IncuCyte SX1^®^ at different times p.i. Our results demonstrated that MAP phagocytosis is pathogen number- and cell density-dependent. Overall, the results showed that infection of 1 × 10^6^ cells at an MOI of 1:10 maintained a stable fluorescence signal of approximately 400 to 500 GCU ⋅ μm^2^/image across 2 h to 4 days p.i. In addition, good correlations between MAP-GFP (logCFUs) estimated in the BACTEC MGIT™ 960 system and fluorescence intensity measured in the IncuCyte SX1^®^ were observed for free MAP-GFP bacilli and from MAP-GFP within infected BOMAC cells, *r* = 0.79 and *r* = 0.78, respectively. In conclusion, real-time fluorescent microscopy enables continuous monitoring of MAP-GFP within infected macrophages.

## 1. Introduction

Bovine paratuberculosis (PTB) or Johne’s disease is a chronic enteritis that affects domestic and wild ruminants and is caused by *Mycobacterium avium* subsp. *paratuberculosis* (MAP) infection. PTB is a major problem for animal health that compromises animal welfare and causes important economic losses to the dairy industry [1]. PTB is an endemic disease in dairy cattle in Europe and North America, with herd prevalence estimates higher than 50% [2,3,4]. Animals are usually infected early in life through the fecal–oral route, but clinical signs including diarrhea and losses in milk production tend to appear when animals are 18 months or older. Once ingested, MAP reaches the ileum and crosses the intestinal mucosa through enterocytes or by binding to fibronectin β1 receptors present on M cells located in Peyer’s patches [5,6,7]. In the submucosa, MAP is phagocytized by sub-epithelial macrophages. MAP has developed several survival mechanisms that allow it to survive inside macrophages, such as preventing the maturation and acidification of the phagosome and its fusion with the lysosome or preventing the presentation of antigens to the immune system [8,9]. PTB is a multifactorial disease, whose outcomes are the result of the interaction between host genetics, environmental, and microbial factors. There are similarities between histopathological lesions and clinical signs that appear in PTB-infected cattle and in human patients with various gastrointestinal inflammatory diseases such as Crohn’s disease (CD) and inflammatory bowel disease (IBD) [10,11,12]. Moreover, MAP has been identified in blood and tissue samples from CD patients [13,14,15], as well as in milk of pregnant women with CD [16]. Additionally, studies support that antimycobacterial antibiotics can induce the remission of pediatric CD [17]. However, a causal association between MAP and CD has not been demonstrated and the potential mechanism by which MAP could cause CD is unknown [18].

Measurement of MAP load within macrophages using a mycobacterial growth inhibition assay (MGIA) is an accurate indicator of host immune control of MAP infection in vitro. MGIAs can be used to understand MAP-host interaction, to compare the virulence of different MAP strains, to understand the role of specific genetic mutations or candidate genes in macrophages function, and to evaluate the efficacy of vaccines or therapeutics by providing a relevant measure of immunogenicity and protection [19,20,21,22]. MGIAs offer a functional measure of the ability of whole blood or blood cell samples to control MAP growth ex vivo.

Estimating MAP bacterial load is challenging due to the organism’s slow growth rate (24+ hour generation time) and heavy clumping. Bacteriological culture in Herrold’s Egg Yolk Medium (HEYM) or Middlebrook 7H10/7H11 supplemented with mycobactin J requires 4–16 weeks for visible colonies to form and cell clumping often underestimates actual cell counts [23]. Automated liquid culture systems such as the BACTEC MGIT™ 960 system track oxygen consumption over time in liquid media, are faster than solid agar and can be used for MAP quantification as the time to detection (TTD) in the BACTEC MGIT™ 960 system inversely correlates with initial viable bacterial load. However, the BACTEC MGIT™ 960 system requires up to 42 days of incubation to yield results, cannot track intracellular bacterial loads within host macrophages in real-time, and requires a host cell lysis step [24]. Novel imaging platforms allow for the real-time monitoring of macrophages and the study of cellular and bacterial dynamics in real time. By capturing and analyzing live cells directly and by collecting both phase contrast and fluorescence images in a high throughput format, live-cell imaging platforms reduce the risk of variations introduced by other methods. For instance, the IncuCyte^®^ system is an advanced, automated imaging platform used for real-time analysis of live cells and microorganisms. When applied to fluorescent microorganisms, the IncuCyte^®^ provides a non-invasive method to quantify fluorescence intensity, which correlates directly with microorganism concentration. Additionally, it allows for the monitoring of fluorescence within infected cells over time without the need of a lysis step or additional steps. Furthermore, the IncuCyte^®^ system allows for the measurement of several 24 or 96 well plates simultaneously, which makes it an invaluable tool for high-throughput studies. The use of the IncuCyte^®^ system has been tested for the quantification of *Mycobacterium tuberculosis* [25] and for the evaluation of antimycobacterial drugs [26]. Additionally, a recent publication by Blay-Benach et al. suggested that this system could also be used to evaluate in vitro macrophage phagocytosis of *Mycobacterium fortuitum* by caprine alveolar macrophages [27]. Given the slow growth of MAP and the prolonged incubation required for conventional culture-based quantification, the development of an automated live-cell fluorescence imaging assay using the IncuCyte SX1^®^ platform to monitor green fluorescence protein (GFP)-expressing MAP (MAP-GFP) in bovine macrophages could be very useful. The objective of the current study was, therefore, the development and optimization of a real-time automated fluorescent method using the IncuCyte SX1^®^ system for measuring MAP-GFP both as free bacilli and within infected bovine macrophages. The use of the IncuCyte SX1^®^ system in combination with MAP-GFP eliminates the need to lyse infected macrophages or wait weeks for slow-growing MAP colonies to appear on agar plates. In summary, the IncuCyte^®^ system might advance MAP research by enabling high-throughput, non-invasive, and continuous real-time monitoring of MAP-GFP and host-macrophage dynamics without the need for destructive endpoint assays.

## 2. Results

### 2.1. Concentration-Dependent Detection of MAP-GFP Fluorescence in the IncuCyte SX1^®^

To evaluate the detection efficiency and linearity of the IncuCyte SX1, fluorescence from ten-fold serial dilutions of MAP-GFP ranging from 3 × 10^7^ to 3 × 10^2^ CFUs were quantified (Figure 1). Quantitative analysis revealed a concentration-dependent decline in the mean count of fluorescent units (Figure 1A).

The highest concentration tested (3 × 10^7^) yielded the maximum detection rate, with 10.25 ± 3.5 mean count of fluorescent units per image. A sharp reduction in detected fluorescence was observed upon the first ten-fold dilution to 3 × 10^6^ CFUs, which exhibited a 2.0 ± 1.41 mean count of fluorescent units per image. Further dilutions to 3 × 10^5^ and 3 × 10^4^ CFUs resulted in minimal signal detection, averaging 0.75 and 0.25 mean count of fluorescent units, respectively. Signal detection was entirely lost at the lowest dilution thresholds of 3 × 10^3^ and 3 × 10^2^ CFUs. Statistical analysis confirmed that the mean count of fluorescent units at the maximum concentration (3 × 10^7^) was significantly higher than all subsequent dilutions (*p* ≤ 0.05). Significant differences were observed only at the 3 × 10^7^ concentration, although a fluorescent signal began to appear at 3 × 10^4^ CFUs. Microscopic evaluation qualitatively validated the quantitative findings (Figure 1B) with well-distributed green, fluorescent signals at the highest concentration (top-left panel). In agreement with the numerical data, a sequential reduction in fluorescent spots was observed across the imaged fields, culminating in a complete absence of visible signal in the lowest concentration panels (bottom panels).

### 2.2. Optimization of Culture Media for IncuCyte SX1^®^ Live-Cell Imaging of MAP-GFP Within BOMAC Cells

BOMAC cells (10^6^ cells/mL) were infected with a single-cell suspension of MAP-GFP at a multiplicity of infection (MOI) of 1:10. After a 2 h incubation, the uninfected and infected cells were replenished with one of the following media: phosphate-buffered saline (PBS), fetal bovine serum (FBS), RPMI-1640, RPMI-1640 without phenol red (PR), and DMEM/F12 with and without 10% FBS supplementation.

RPMI-1640 was originally designed specifically for the culture of human lymphocytes and immune cells (such as macrophages and monocytes, which are frequently used in infection assays). DMEM/F12 is a 1:1 combination of Dulbecco’s Modified Eagle Medium and Ham’s F-12. It is a nutrient-rich formulation containing a much higher concentration of amino acids, vitamins, and glucose than standard RPMI or basic DMEM. Adding FBS supplies essential growth factors, hormones, and lipids that keep host cells alive and metabolically active over multi-day infection periods without prematurely killing the host cells. Fluorescence was quantified using the IncuCyte SX1^®^ system, analyzed with the IncuCyte^®^ controller version 2022A and expressed as mean total integrated fluorescence intensity (GCU × μm^2^/Image). Among all media tested, only DMEM/F12, DMEM/F12 supplemented with FBS, and FBS-supplemented RPMI-1640 yielded significant differences between infected and uninfected cells (*p* = 0.035, *p* = 0.034, and *p* = 0.029, respectively) (Figure 2). RPMI-1640 supplemented with FBS was selected for future experiments, as it is the recommended media for BOMAC growth and because the highest significant differences in mean total integrated fluorescence intensity were observed between uninfected and infected BOMAC cells in this medium.

### 2.3. Optimization of MOI for IncuCyte SX1^®^ Live-Cell Imaging of MAP-GFP Within MDMs and BOMAC Cells

To evaluate the relationship between bacterial concentration and fluorescence quantification, MDMs and BOMAC cells were inoculated by triplicate with varying MOIs, and after 2 h of infection the resulting total integrated fluorescence intensity (mean GCU × μm^2^/Image) was measured in the IncuCyte^®^ system (Figure 3).

The MDMs used represent three independent biological replicates, derived from blood samples collected from three individuals. The highest fluorescence signal was observed at a MOI 1:10 with mean total integrated fluorescence intensity of approximately 154.7 ± 49.2 units (Figure 3A). A marked reduction in fluorescence was observed at a MOI of 1:1 (53.7 units), while further dilutions ranging from 1:0.1 down to 0.00001 resulted in a complete loss of detectable fluorescence signal. Statistical analysis confirmed that the fluorescence at the MOI 1:10 was significantly elevated compared to all lower MOI conditions (*p* < 0.001). This quantitative trend was observed by representative images (bright phase-green fluorescence) (Figure 3B). MDMs infected at MOI 1:10 exhibited robust green, fluorescent signal (top-left panel). A clear reduction in the intensity of fluorescence was observed with the MOI of 1:1 (top-right panel), culminating in a total absence of green fluorescence signal at the lowest MOI of 1:0.1 and 1:0.01 (bottom panels).

A refined MOI gradient was assessed in BOMAC cells ranging from 1:100 down to 1:0.05, alongside an uninfected control (Figure 3C). Maximum fluorescence was detected at the highest tested MOI of 1:100, producing a mean total integrated fluorescence intensity of approximately 1306 GCU × μm^2^/Image. The signal intensity decreased as the MOI was diluted to 1:50 (659 GCU × μm^2^/Image) and 1:10 (327 GCU × μm^2^/Image). Below the MOI of 1:10, fluorescence maintained low levels (∼124 − 304 GCU × μm^2^/Image) that closely mirrored the background signal observed in the uninfected control group (166 GCU × μm^2^/Image). Comparatively, the uninfected control exhibited a statistically significant reduction in mean total integrated fluorescence intensity when measured against experimental MOIs from 1:100 down to 1:0.1 (*p* ≤ 0.0000001). Every single comparison between MOI 1: 100 and MOI 1:50 against all lower MOI levels and the uninfected group yields a *p*-value <10^−7^. Collectively, these results suggested that green fluorescence intensity directly correlates with the MOI across both MDMs and BOMAC cells after 2 h of infection. In MDMs and BOMAC cells, green fluorescence intensity similarly peaked at the highest tested MOI (1:10 and 1:100, respectively) and progressively decreased as the bacterial concentration was diluted.

### 2.4. Optimization of Cell Concentration and Time-Course Quantification of MAP-GFP Fluorescence Within Infected BOMAC Cells

To illustrate the IncuCyte SX1^®^ platform ability to carry out long-term imaging, total integrated fluorescence intensity was tracked over 7 days across varying cell densities (5 × 10^4^, 1 × 10^5^, and 1 × 10^6^ cells) and MOIs (1:10 and 1:100) (Figure 4). As shown in Figure 4A, when 10^6^ cells were infected at a MOI 1:100 (blue line), the fluorescence signal increased significantly starting day 4 reaching its maximum by day 7 (6 × 10^5^ GCUμm^2^/Image). Microscopic observation revealed that this increase in fluorescence was accompanied by a sudden dispersion of localized GFP signal reflecting host cell death at this high MOI (Supplementary Figure S1). In contrast, green fluorescence in lower cells’ density conditions (1 × 10^5^ and 5 × 10^4^ cells) infected at a MOI 1:100 remained near baseline throughout the 7-day period. Rescaling the y-axis by excluding MOI 1:100 (10^6^ cells/mL) revealed a stable fluorescence signal from 2 h to 4 days p.i. for 10^6^ cells/mL infected with a MOI of 1:10 (Figure 4B). Figure 4C only shows early kinetics. Cells (1 × 10^6^) infected with a MOI 100 (blue line) display a steady, gradual increase in mean total integrated fluorescence intensity from 2 h (∼500 GCU ⋅ μm^2^/Image) up to day 3 (∼1250 GCU ⋅ μm^2^/Image), before beginning its sharp increase at day 4 (∼2800), reflecting cell death. However, infection of BOMAC cells (1 × 10^6^ cells/mL) with a MOI 1:10 (pink line) maintained a stable baseline of ∼400–500 GCU ⋅ μm^2^/Image across days 0 to 4 days pi. suggesting that these might be the optimal assay conditions. All other conditions remain flat near baseline during these initial 4 days.

Phase area confluence percentages were measured in the IncuCyte SX1^®^ and compared between time points and MOIs using ANOVA with Tukey correction. Phase area confluence percentages remained unaffected by the different MOI treatments (MOI 1:100 and 1:10) up to 4 days post-infection (*p_adj_* > 0.05); however, by day 5, a significant difference in cell confluence percentages between MOI 10 and MOI 100 emerged exclusively at the highest cell concentration and MOI (MOI, 1:100, 1 × 10^6^ cells/mL, and *p_adj_* = 0.011). At this cell concentration, a MOI of 100 means we are infecting the cells with 1 × 10^8^ bacteria, whereas an MOI of 10 means we are infecting the cells with 1 × 10^7^ bacteria. At lower cell concentrations (5 × 10^4^), the total number of bacteria added even at MOI 100 is much smaller (5 × 10^6^), which may not be enough to cause cytotoxicity over a 5-day period.

### 2.5. A Comparative Analysis of the IncuCyte^®^ Platform and BACTEC MGIT™960 Liquid Culture System

To examine whether MAP-GFP fluorescence correlated with MAP CFU numbers, we compared fluorescence intensity versus log CFUs. Serial 10-fold dilutions of a suspension of MAP-GFP bacilli (McFarland standard of 1) were quantified by triplicate in the IncuCyte SX1^®^ and in the BACTEC-MGIT™ 960 system (Figure 5A). We found a good correlation between the mean count of fluorescence units obtained in the IncuCyte SX1^®^ and logCFUs calculated with BACTEC MGIT™ 960 system (*r* = 0.79, 95% CI = [−0.07371659–0.97522596]), with an incremental increase in fluorescence translating to an increase in the number of bacilli. To assess the robustness of the correlation, we excluded the highest concentration point, which still yielded a strong linear relationship (*r* = 0.873). Additionally, Spearman rank correlation analysis, less sensitive to extreme values, confirmed a strong correlation (*ρ* = 0.986).

In addition, mean total fluorescence intensity of BOMAC cells (10^6^ cells/mL) infected with MAP-GFP at several MOIs (1:100, 1:50, 1:10, 1:5, 1: 0.1, 1:1, and 1: 0.05) in triplicate was quantified in the IncuCyte SX1^®^ and compared to estimated logCFUs calculated in the BACTEC MGIT™ 960 system (Figure 5B). There was also a good correlation between mean fluorescence integrated intensity measured in the IncuCyte SX1^®^ and logCFUs estimated in the BACTEC MGIT™ 960 system (*r* = 0.78, 95% CI = [0.08237824–0.96691230]).

## 3. Discussion

Previous studies have used a variety of assays to study MAP phagocytosis, including plate counting, fluorescence microscopy, flow cytometry, and spectrofluorometry. However, technical and practical limitations are associated with all these methods, such as low throughput, single end point readouts, and often subjective quantification. Fluorescence microscopy and flow cytometry techniques have been used to visualize and quantify phagocytosis of *E. coli* bioparticles by non-adherent phagocytic cells or macrophage cell lines [28]. However, primary macrophages can be stressed when detached from a plate to be run on a flow or imaging flow cytometer and the analysis is of low throughput and potentially open to operator bias. In addition, these methods are time-consuming and require a cell fixation step, which can potentially disrupt macrophages. By using real-time and live-cell assays with fluorescently labeled bacteria, fluorescence intensity can be tracked directly and continuously using automated live-cell imaging platforms such as the IncuCyte^®^ system. To our knowledge, this study is the first to monitor MAP-GFP using live-cell fluorescence microscopy.

Our study demonstrated that MAP phagocytosis is pathogen number- and cell density-dependent. Infecting BOMAC cells (10^6^ cells/mL) with MAP-GFP at an MOI of 1:10 (3 × 10^7^ CFUs) yielded stable fluorescence in the IncuCyte^®^ system over the first 4 days p.i. This aligns with previous live-cell imaging studies utilizing the IncuCyte^®^ system, which established that low MOIs (e.g., 1:10) maintained host cell integrity and avoided fluorescence spikes caused by high infection doses of *Mycobacterium tuberculosis* (*Mtb*) such as a MOI 1:100 [29]. Our results are also consistent with previous studies showing that MAP is cytotoxic to macrophages at high doses [8]. The sharp increase in fluorescence observed at day 4 post-infection (p.i.) at a MOI of 1:100 likely reflects host cell death. Adding a high bacterial load (MOI 100) on top of high density (1 × 10^6^ cells/mL) generates rapid nutrient depletion, accumulation of metabolic waste, and host cell stress, rounding up, detachment, and/or cell death. By day 5, this manifests as a clear divergence in cell confluence percentages between MOI 10 and MOI 100. Establishing that cell confluence remained stable during the first 4 days ensured that any observed differences were not confounded by premature host cell death, while highlighting the delayed cytotoxicity specific to high-cell density, high-MOI conditions by day 5. Tracking host cell confluence using live-cell imaging systems like IncuCyte is crucial to track monolayer integrity and assess potential cytopathic effects induced by bacterial infection over time. This is supported by representative images captured on day 5 p.i. using a high bacterial load (MOI 100) and a high cell density (1 × 10^6^ cells/mL) (Supplementary Figure S1). At this time point, the software segmentation mask selectively tracks large, interconnected cell clusters. However, these intact cells are surrounded by a massive background population of small, round, and dark spheres. These structures filling the background are highly consistent with cellular fragmentation, apoptotic bodies or advanced cell debris. They do not display the healthy, flattened, and adherent morphology of a typical healthy monolayer.

Although previous studies used cell densities of 10^4^ or 10^5^ cells per well [25,27], our study demonstrated that the infection of BOMAC cells (10^6^ cells/mL) with MAP-GFP at a MOI 1:10 gave consistent results over 4 days pi. Furthermore, the IncuCyte SX1^®^ system could detect differences in fluorescence in BOMAC cells infected with different concentrations of MAP-GFP, which correlated with logCFUs estimated in the BACTEC MGIT™ 960 system. Other studies have evaluated phagocytosis of *Mycobacterium fortuitum* by alveolar macrophages using a pH-dependent dye and also obtained good correlation values between fluorescence quantification and TTDs obtained from the BACTEC MGIT™ 960 system [27].

The most relevant system to study phagocytosis and intracellular survival of MAP would be macrophages isolated from the intestinal tract. However, this is not a practical model because of technical difficulties in culturing mononuclear phagocytes from this site. A commonly used alternative approach is to use peripheral blood monocytes. However, variations in donor origin can introduce phenotypic heterogeneity that may influence intracellular bacterial survival and immune responses. In our study, we also used BOMAC cells. While cell lines provide consistent and reproducible models, they may not fully capture the phenotypic signaling, receptor expression, and donor variability present in primary host cells during infection.

Published live-cell kinetic studies utilizing platforms like IncuCyte^®^ rely on non-normalized total integrated intensity to capture cell population outcomes [26,27]. As a cumulative metric, total integrated fluorescence intensity reflects overall infection dynamics rather than a per-cell measurement. Sartorius recommends total integrated fluorescence intensity (often measured in green calibrated units per image or well) as a primary metric for live-cell assays because it provides a comprehensive, highly sensitive, and biologically complete measurement of fluorescence. Specifically, this metric captures both signal extent and brightness, is well-suited for real-time kinetics, offers a wider dynamic range, and accommodates cellular heterogeneity. Because cell cultures inherently vary in size, shape, and expression capacity, integrating all pixel values across the region of interest—rather than averaging them—smooths out cell-to-cell variability and provides a robust, reliable quantitative signal for the entire well. This allows detecting subtle or early-stage responses that might be missed by threshold-based object counts. In our study, phase-contrast imaging demonstrated that cell monolayer confluence did not vary significantly across triplicate wells and treatment groups up to 4 days p.i. Because variations in cell confluence across wells and MOIs were not statistically significant up to 4 days p.i., raw total integrated intensity data were analyzed directly without normalizing to cell confluence (Figure 4). In addition, quantification of 25 high-resolution images per well provided a representative measurement of the overall fluorescence signal for statistical comparisons.

Despite its utility for real-time monitoring of intracellular bacteria, fluorescence microscopy presents some limitations. It should be noted that the microscopic enumeration of acid-fast bacilli could reflect both viable and non-viable bacilli, whereas the BACTEC method quantifies metabolically active bacilli. This distinction is particularly critical when evaluating antimicrobial interventions: if a drug or treatment exerts a bactericidal effect without inducing full cell lysis, stable GFP molecules can linger within non-viable bacterial remnants or extracellular debris. As a result, fluorescence spikes may overestimate bacterial survival, yielding false-negative assessments of treatment efficacy. Our results showed that the IncuCyte SX1^®^ data strongly correlated with the metrics from the BACTEC MGIT 960, which proves that the fluorescent signal on the IncuCyte SX1^®^ is primarily driven by living bacteria rather than an accumulation of dead bacterial debris or persistent background fluorescence. Although the MAP suspension used for cell infection was passed 20 times through a 27-gauge needle to remove bacterial clumps, we cannot exclude the possibility that residual clumping introduced some variability in the IncuCyte SX1^®^ data, thereby increasing standard deviations, particularly at higher concentrations.

While the IncuCyte^®^ Live-Cell Analysis System offers major advantages for real-time kinetic imaging compared to traditional endpoint assays, it also comes with some technological and practical limitations, including lower magnification, bottleneck for multiple users, and data storage requirements. Because the system is designed to scan entire microplates, it typically relies on lower-magnification objectives, and it cannot replace high-resolution confocal microscopes for detailed internal organelle tracking. Because kinetic experiments require monitoring the same plates continuously over days, a single IncuCyte^®^ unit can quickly become a bottleneck if multiple lab members need to run long-term assays simultaneously. Continuous high-content imaging—especially across multiple fluorescent channels and numerous wells—generates massive datasets. Processing and storing these files requires robust infrastructure.

## 4. Materials and Methods

### 4.1. Ethic Statement

The study is reported in accordance with ARRIVE guidelines format 2 (https://arriveguidelines.org, accessed on 28 July 2026). Blood samples were collected by trained personnel and in accordance with good veterinary practices and following the European Guidelines for the Care and Use of Animals for Research Purposes (2012/63/EU). The blood extraction procedure was approved by the Ethics Committee of NEIKER (Organo encargado de bienestar animal y comité evaluador; OEBA-CE) and authorized by the Departamento de Ganadería de la Diputación de Alava (approval number: 4190-2022; approval date: 28 November 2022).

### 4.2. Bacterial Strains and Cell Line

The K10 strain of MAP, a sequenced and laboratory-adapted isolate recovered from a clinical case of PTB, was obtained from the American Type Culture Collection (ATCC, Manassas, VA, USA). MAP-GFP was kindly provided by Dr. Barletta (University of Nebraska, US). The BOMAC cell line, an SV-40 transformed bovine peritoneal macrophage cell line, was kindly provided by Dr. Stabel (USDA, Ames, IA, USA) and maintained in RPMI-1640 medium with 20 mM L-glutamine (Corning, NY, USA) supplemented with 10% heat-inactivated FBS (Corning, NY, USA) and 1% penicillin/streptomycin (Corning, NY, USA) as previously described [30]. Information regarding the BOMAC cell line has been deposited in Cellosaurus (https://www.cellosaurus.org/CVCL_B6PF, accessed on 28 July 2026).

### 4.3. Bacterial Culture and Preparation of Bacterial Suspensions

MAP K10 and MAP-GFP strains were grown in T25 tissue culture flasks at 37 ± 1 °C in 10 mL of Middlebrook 7H9 broth (Difco Laboratories, Detroit, MI, USA) supplemented with 10% (*v*/*v*) oleic acid-albumin-dextrose-catalase (Becton, Dickinson and Company, Franklin Lakes, NJ, USA), 0.05% (*v*/*v*) Tween-80 (Sigma-Aldrich, St Louis, MO, USA) and 2 mg l^−1^ of Mycobactin J (Allied Monitor Inc., Fayette, MO, USA) for 3 weeks at 37 °C ± 1 °C. Kanamycin (Sigma-Aldrich, St. Louis, MO, USA) was added to the media at 50 μg/mL for growing the MAP-GFP strain as previously described [31]. Bacterial cells were harvested by centrifugation at 2000× *g* for 20 min in a Beckman Coulter Allegra X-12 centrifuge (Beckman Coulter, Inc., Brea, CA, USA). Bacterial pellets were washed three times with sterile HBSS, resuspended in 2 mL of HBSS, and the resultant suspension was passed 20 times through a 27-gauge needle, and large aggregates were allowed to settle. After 5 min, an aliquot was taken from the top half of the bacterial suspension and diluted in HBSS to a McFarland standard of 1 (3 × 10^8^ CFUs/mL) with a Densimat (bioMerieux, Marcy l’Étoile, France).

### 4.4. Preparation of MAP-GFP Dilutions and IncuCyte^®^ Fluorescence Quantification

Serial 10-fold dilutions of a suspension of MAP-GFP bacilli (McFarland standard of 1) were prepared in HBSS with vortexing between dilution steps. Then, 100 μL of each dilution (3 × 10^7^, 3 × 10^6^, 3 × 10^5^, 3 × 10^4^, 3 × 10^3^, and 3 × 10^2^ CFUs) was added to a 24-well plate and fluorescence was recorded in the IncuCyte^®^ SX1 (Sartorius, Göttingen, Germany), housed inside an incubator at 37 °C with 5% CO_2_. Twenty-five images per well from three replicates were taken and analyzed using the adherent cell-by-cell module of the IncuCyte^®^ controller version 2022A. Green channel acquisition time was 300 ms, and phase contrast was left as default. Green channel background noise was subtracted with the Top-Hat method of background non-uniformity correction with a radius of 10 μm, a threshold of 1 green calibrated unit (GCU), and an edge sensitivity of −30. The mean count of fluorescent units was used as a measure of fluorescence quantification.

### 4.5. Bovine MDM Preparation

MDMs were prepared as previously described [20]. Briefly, fifteen milliliters of peripheral blood were drawn from the tail vein of three adult healthy Holstein cows from the same farm in Alava (Basque Country, Spain) into heparinized Vacutainer tubes (Becton, Dickinson and Company, Sparks, MD, USA) and diluted 1:2 in Hanks balanced salt solution (HBSS). Leucosep tubes were filled with 15 mL of Ficoll-Paque (1.084 g/cm^3^) (GE Healthcare, Uppsala, Sweden) and centrifuged at 1000 rpm for 30 s at room temperature. Subsequently, the diluted blood was overlaid on the top of the Ficoll-Paque and centrifuged at 800 *g* for 15 min at room temperature. The plasma layer was removed and the cell interphase containing peripheral blood mononuclear cells (PBMCs) was collected and transferred to a clean tube. PBMCs were washed twice in HBSS and centrifuged at 400 *g* for 10 min to remove platelets. Supernatants were aspirated and the purified PBMCs were resuspended in RPMI-1640 supplemented with 20 mM l-glutamine, 10% heat-inactivated bovine serum (Lonza, Spain), 100 U mL^−1^ penicillin G, and 100 mg mL^−1^ streptomycin sulfate (Lonza, Spain). PBMCs were cultured by triplicate at a concentration of 1 × 10^6^ cells/mL into 24-well flat clear bottom tissue-culture-treated plates and incubated at 37 °C in a humidified 5% CO_2_ incubator at 37 °C for 2 h. Non-adherent cells were removed by washing, and adherent cells were incubated in fresh medium for 7 days at 37 °C to allow differentiation to MDMs.

### 4.6. Optimization of Culture Media for IncuCyte^®^ Live-Cell Imaging of MAP Within BOMAC Cells

BOMAC cells were seeded at a concentration of 10^6^ cells/mL into 24-well plates and infected with a single-cell suspension of MAP-GFP at MOI of 1:10. After 2 h, the supernatant was removed, and uninfected and MAP-GFP-infected BOMAC cells were washed three times with HBSS. Each wash includes complete aspiration of the supernatant to ensure the removal of loosely adherent or floating bacteria. PBS, FBS (Cytiva, Marlborough, MA, USA), RPMI-1640 (Corning, Waltham, MA, USA), RPMI-1640 supplemented with 10% FBS, RPMI-1640 without PR (w/o PR), RPMI-1640 w/o PR supplemented with 10% FBS, DMEM/F12 (Cytiva, Marlborough, MA, USA), and DMEM/F12 supplemented with 10% FBS were added to MAP-GFP-infected and -uninfected cells. The plates were transferred into the IncuCyte SX1^®^ system, which was housed inside an incubator at 37 °C. Fluorescence was subsequently quantified as described above, with mean total integrated intensity (GCU × μm^2^/Image) serving as a measure of intracellular MAP load.

### 4.7. Optimization of MOI for IncuCyte^®^ Live-Cell Imaging of MAP-GFP-Infected BOMAC Cells and MDMs

BOMAC cells and MDMs were seeded by triplicate at a concentration of 10^6^ cells/mL into 24-well plates and incubated at 37 °C in an incubator with 5% CO_2_. The following day, the BOMAC cells were infected with MAP-GFP at various MOI (1:100, 1.50, 1:10, 1:5, 1:1, 1:0.1, and 1:05). Bovine MDMs were infected at MOIs of 1:10, 1:1, 1:0.1, 1:0.01, 1.0.001, 1:0.0001, and 1:0.00001. After 2 h, the supernatant was removed, the cells were washed three times with HBSS and fresh RPMI-1640 supplemented with 10% FBS was added to the cells. Then, the fluorescence was read in the IncuCyte SX1^®^ system as described above and expressed as mean total integrated intensity (GCU × μm^2^/Image).

### 4.8. Optimization of Cell Concentration and Time-Course Quantification of MAP-GFP Fluorescence Within Infected BOMAC Cells

BOMAC cells were seeded in triplicate in 24-well plates at a concentration of 10^6^, 10^5^ and 5 × 10 ^4^ cells/mL into 24-well plates and were infected with MAP-GFP at MOI of 1:10 and 1:100. At 2 h post-infection, the supernatant was removed, the cells were washed with HBSS and fresh RPMI-1640 supplemented with 10% FBS was added to the cells. Then, the plate was placed in the IncuCyte SX1 ^®^ and incubated at 37 °C with 5% CO_2_. Fluorescence signal was measured at 2 h p.i. and every day for 7 days in the IncuCyte SX1^®^, as described above, and expressed as mean total integrated intensity (GCU × μm^2^/Image).

### 4.9. A Comparative Analysis of the IncuCyte^®^ Platform and BACTEC MGIT™ 960 Liquid Culture System for MAP Quantification

Serial 10-fold dilutions of a suspension of MAP-GFP bacilli (McFarland standard of 1) were prepared in HBSS with vortexing between dilution steps. Then, 100 μL of each dilution was added to a 24-well plate (3 × 10^7^, 3 × 10^6^, 3 × 10^5^, 3 × 10^4^, 3 × 10^3^, and 3 × 10^2^ CFUs) in triplicate, and fluorescence was recorded in the IncuCyte^®^ (Sartorius, Göttingen, Germany). In addition, 100 μL of each dilution was quantified in the BACTEC MGIT™ 960 (Becton, Dickinson, and Company, Sparks, MD, USA) system as previously described [24]. BOMAC cells were seeded in triplicate at a concentration of 10^6^ cells/mL into 24-well plates. The following day, the cells were infected with MAP-GFP at seven MOIs (1:100, 1:50, 1:10, 1:5, 1:1, 1: 0.1, and 1: 0.05). At 2 h post-infection, the supernatant was removed, the cells were washed with HBSS and fresh RPMI-1640 supplemented with 10% FBS was added. One of the plates was placed inside the IncuCyte SX1^®^ and the fluorescence was recorded as total integrated fluorescence intensity. The duplicate plate was lysed at 2 h p.i. by vigorous pipetting with 0.5 mL of 0.1% Triton X-100 (Sigma-Aldrich, St. Louis, MO, USA) in sterile water for 5 min. Supplemented Mycobacteria Growth Indicator tubes (MGITs) (Becton, Dickinson, and Company, Sparks, MD, USA) were inoculated 0.5 mL of the cell lysates and the tubes were incubated at 37 °C ± 2 °C for up to 42 days in a BACTEC MGIT™ 960 instrument. The earliest instrumental indicator of positivity (time to detection, TTD) for each tube was recorded. The predicted number of bacteria in each positive tube was calculated using standard curves that relate TTD (in days) to the estimated log CFUs as previously described [24].

### 4.10. Statistical Analysis

Statistical comparisons between two groups were performed using Student’s *t*-test, while comparisons involving more than two groups were performed using one-way ANOVA followed by Tukey’s multiple-comparison test considering adjusted *p*-values ≤ 0.05 as significant. Prior to statistical analysis, the assumptions of normality and homogeneity of variances were confirmed by Shapiro–Wilk test. Correlations between log CFUs estimated in the BACTEC MGIT™ 960 system and fluorescence signal measured in IncuCyte SX1^®^ were assessed by Pearson correlation test.

## 5. Conclusions

Overall, the study demonstrates that MAP-GFP-associated fluorescence within infected macrophages can be monitored longitudinally using the IncuCyte SX1^®^ platform. Our key findings demonstrated the following: (1) a stable fluorescence signal (~400–500 GCU) can be maintained in 1 × 10^6^ cells at an MOI of 1:10 across 2 h to 4 days p.i. and (2) IncuCyte SX1^®^ fluorescence intensity strongly correlates with BACTEC MGIT™ 960 system log CFU estimates (*r* = 0.79 for free bacilli; *r* = 0.78 for intracellular bacilli), establishing real-time live-cell microscopy as a highly reliable method for monitoring mycobacterial infection dynamics.

## Figures and Tables

**Figure 1:**
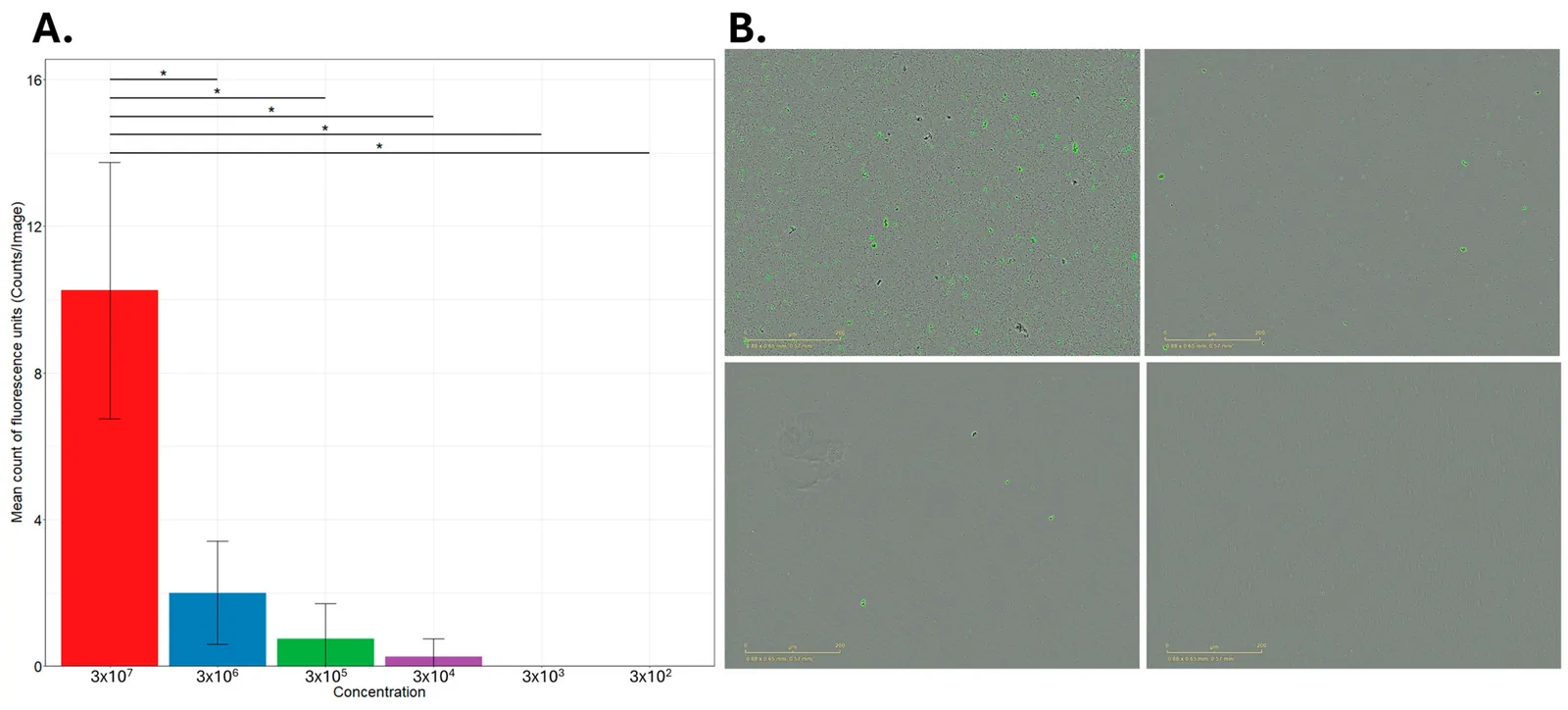
**Quantification of MAP-GFP bacilli in the IncuCyte SX1^®^**. (**A**) Mean count of fluorescence units of each dilution of MAP-GFP. Each bar and whisker represents the mean and the standard deviation, respectively. Significant differences between dilutions of MAP-GFP are indicated by an asterisk (*p* ≤ 0.05). (**B**) Representative (bright phase-green fluorescence) images (20×) of MAP-GFP at concentration 3 × 10^7^, 3 × 10^6^, 3 × 10^5^, 3 × 10^4^ CFUs, from left to right, up to down. Images are from one representative image of three independent experiments.

**Figure 2:**
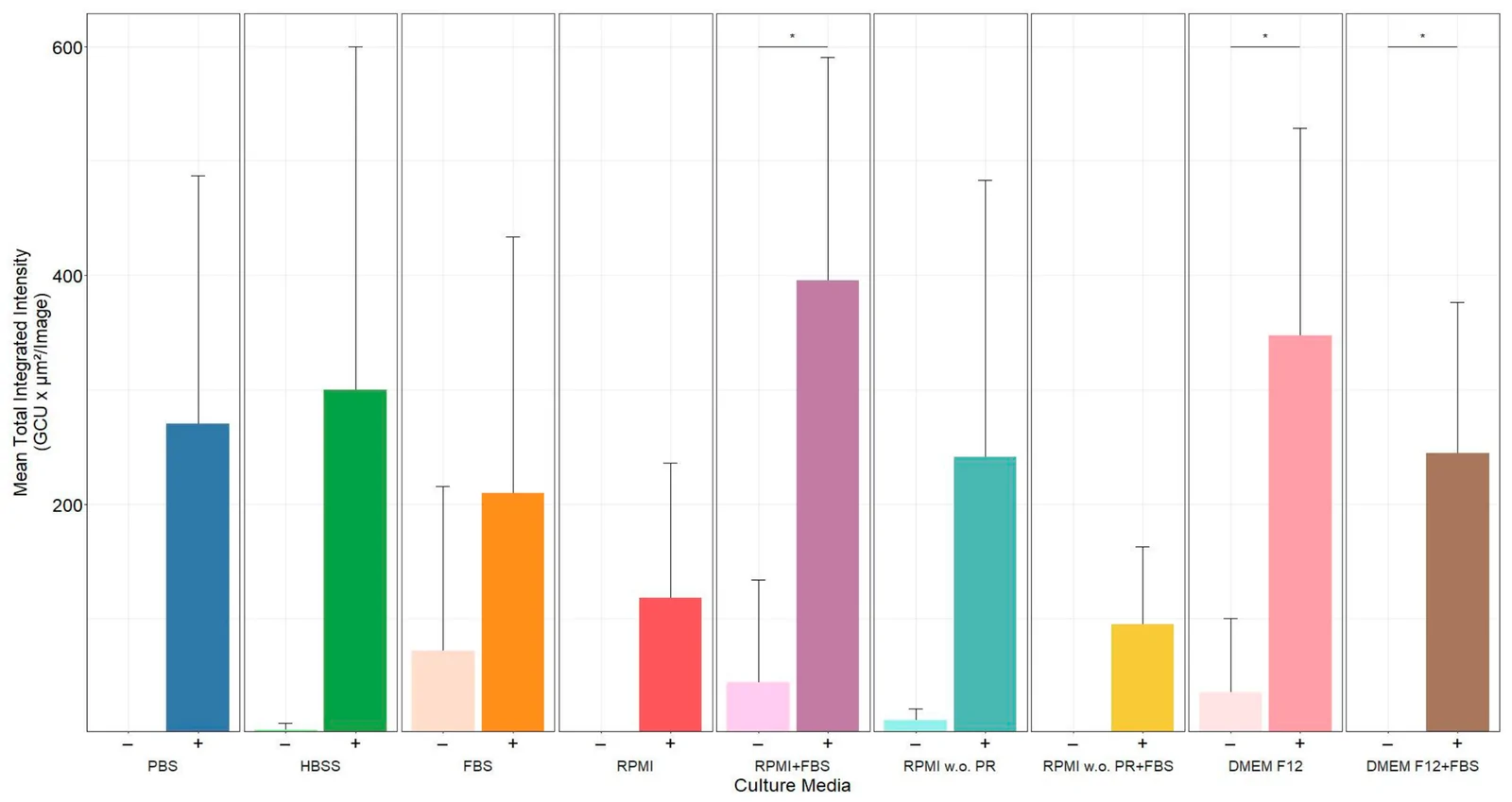
**Mean total integrated fluorescence intensity in BOMAC cells infected with MAP-GFP across culture media.** Comparison of mean total integrated green fluorescence intensity (GCU × μm^2^/image ± SD) between uninfected (−) and infected BOMAC cells (+) across various media: PBS, HBSS, FBS, RPMI-1640 (±FBS/Phenol Red), DMEM F12, and DMEM F12 + FBS. Significant differences between uninfected and infected conditions were observed in RPMI + FBS, DMEM F12, and DMEM F12 + FBS and are indicated by an asterisk (*p* ≤ 0.05).

**Figure 3:**
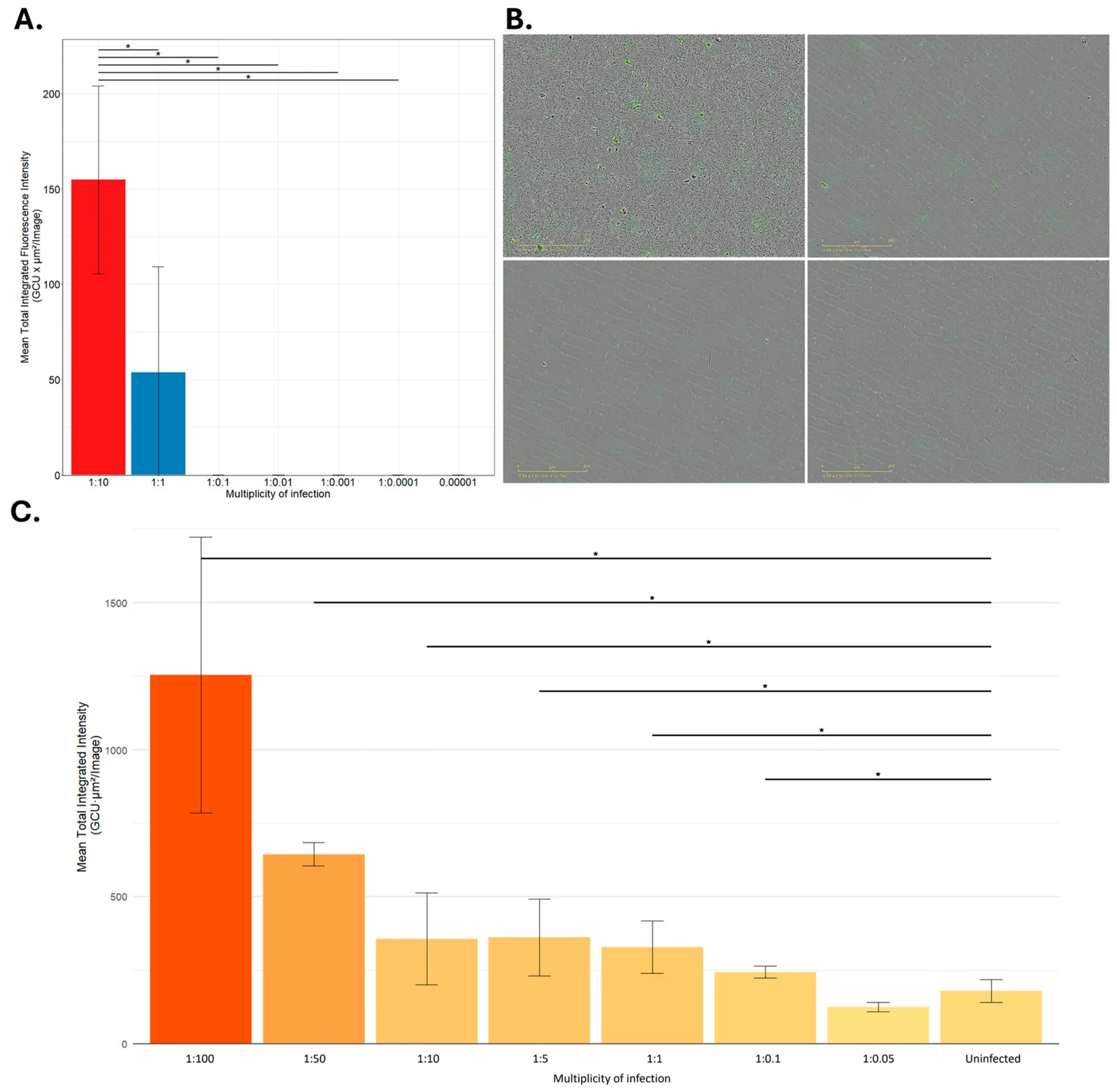
**Mean total integrated fluorescence intensity at 2 h p.i. in MDMs and BOMAC cells infected with MAP-GFP at different MOIs**. (**A**) Histogram representing mean total integrated fluorescence intensity in MDMs infected with MAP-GFP at different MOI (1:10, 1:1, 1:0.1, 1:0.01, 1:0.001, 1:0.0001, 1:0.00001). Each bar and whisker represents the mean and the SD, respectively. Significant differences are indicated by an asterisk (*p* ≤ 0.05). (**B**) Representative images (bright phase-green fluorescence) (20×) of MDMs infected with MAP-GFP at MOIs 1:10, 1:1, 1:0.1, and 1:0.01, from left to right up to down. (**C**) BOMAC cells infected with MAP-GFP at different MOIs (1:100, 1:50, 1:10, 1:5, 1:1, 1:0.1, 1:0.05). Each bar represents the mean total integrated intensity of each MOI. Asterisks represent significant differences between MOIs and the control (*p* < 0.001).

**Figure 4:**
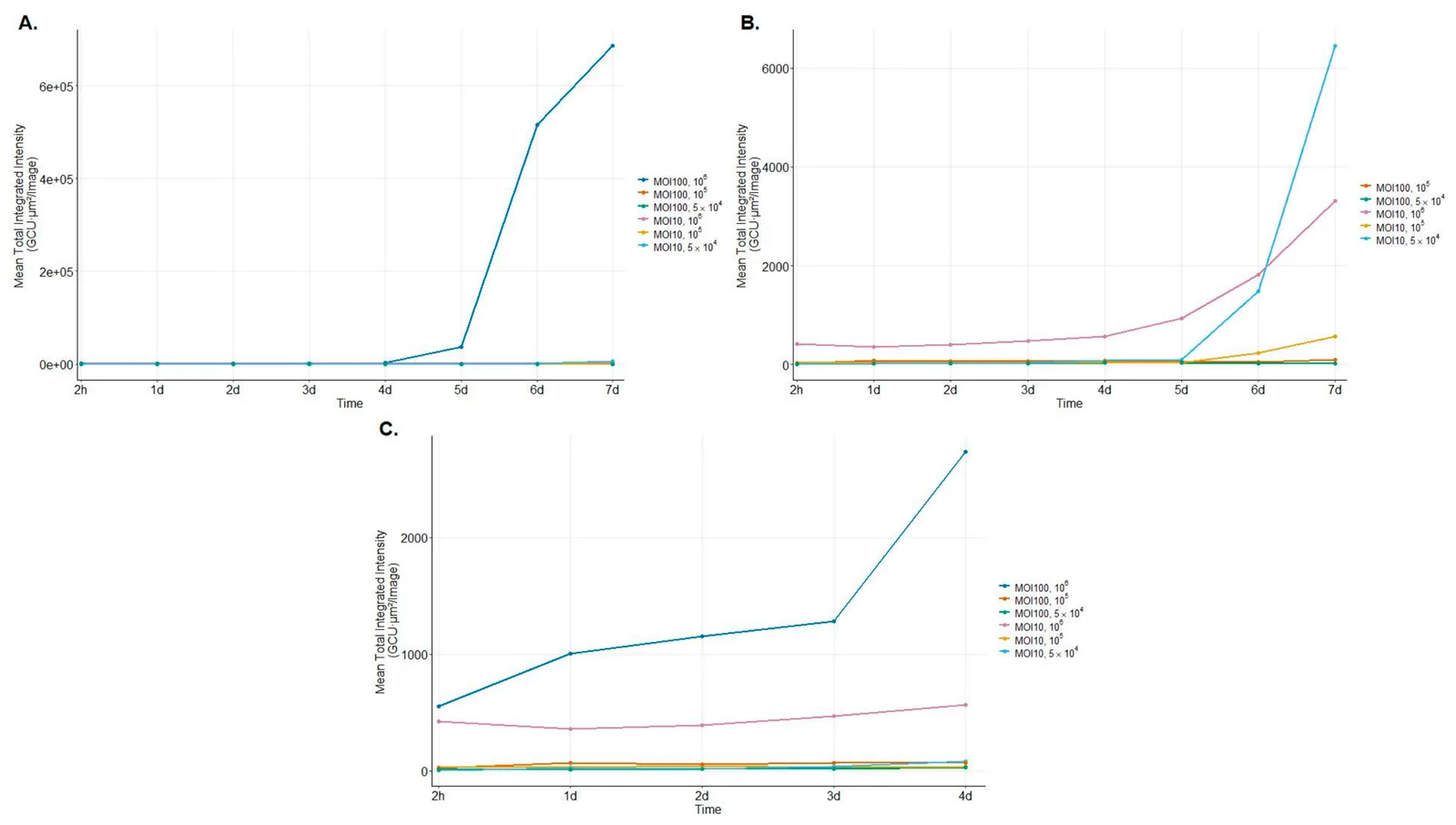
**Longitudinal kinetics of MAP-GFP infection in BOMAC cells across different cell densities (10^6^, 10^5^, 5** × **10^4^ cells) and MOIs (1:10 and 1:100) over 7 days p.i.** (**A**) Mean total integrated green fluorescence intensity (GCU × μm^2^/image) measured over 7 days post-infection. A strong signal surge was observed at day 4 in the highest-cell density/highest-MOI condition (MOI 1:100, 10^6^ cells/mL; blue line) due to host cell death. (**B**) Rescaled view of the 7-day kinetic profile excluding MOI 1:100 with 10^6^ cells/mL. Conditions including MOI 1:10 with 10^6^ cells/mL (pink line) and MOI 1:10 with 5 × 10^4^ cells/mL (blue line) showed signal increases starting after day 4 and 5 suggesting host cell lysis. (**C**) Mean total integrated green fluorescence intensity (GCU × μm^2^/image) measured over 4 days p.i. Infection of 10^6^ cells/mL at an MOI of 1:10 (pink line) maintained a stable baseline (∼400–500 GCU × μm^2^/image), representing the optimal window for assessing intracellular bacterial load prior to significant host cell destruction.

**Figure 5:**
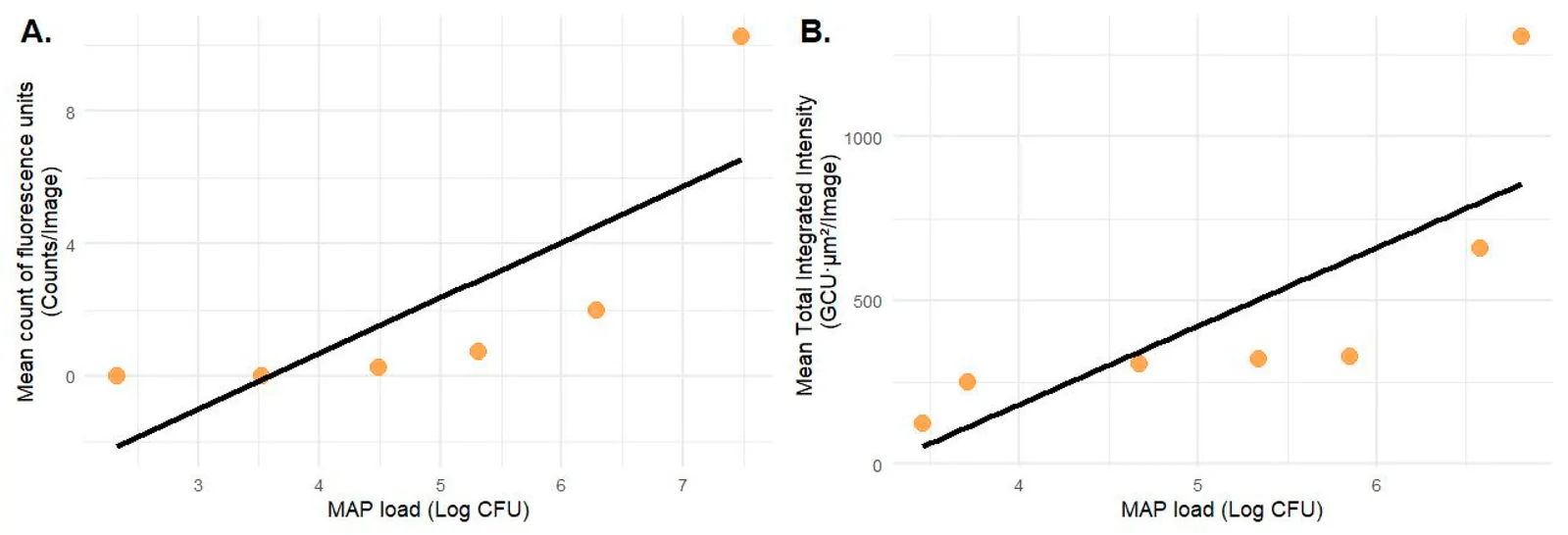
**Correlation between MAP-GFP measured by live-cell imaging in the IncuCyte SX1^®^ and logCFUs calculated with the BACTEC MGIT™ 960 system.** (**A**). Seven 10-fold dilutions of a suspension of MAP-GFP bacilli (McFarland standard of 1) were quantified by triplicate in the IncuCyte SX1^®^ and BACTEC-MGIT™ 960 system. A good correlation between mean fluorescence signal counts obtained in the IncuCyte^®^ and CFUs calculated with BACTEC-MGIT™ 960 systems (*r* = 0.79, 95% CI = [−0.07371659–0.97522596]) was obtained. (**B**) Total integrated fluorescence intensity of BOMAC cells (10^6^ cells) infected with MAP-GFP at seven MOIs (1:100, 1:50, 1:10, 1:5, 1:1, 1: 0.1, and 1: 0.05) in triplicate was compared to logCFUs calculated with the BACTEC MGIT™ 960 system. A good correlation between mean total integrated fluorescence intensity (GCU × μm^2^/image) measured in the IncuCyte^®^ and logCFUs estimated in the BACTEC MGIT™ 960 system was obtained (*r* = 0.78, 95% CI = 0.08237824–0.96691230]). Each time point is the mean of three replicates.

## Data Availability

The original contributions presented in the study are included in the article/Supplementary Material; further inquiries can be directed to the corresponding author.

## Supplementary Materials

The following supporting information can be downloaded at https://www.mdpi.com/article/10.3390/ijms27219640/s1.
