# Supplementary material for: Real-Time Automated Fluorescence Imaging Assay for Quantifying GFP-Expressing Mycobacterium avium subsp. paratuberculosis
Source: Int J Mol Sci. 2026 Oct 28;27(21):9640. doi: 10.3390/ijms27219640 (PMC13763567; doi:10.3390/ijms27219640)

Supplementary Figure S1. Representative phase-contrast images captured on day 5 p.i. of MAP-infected BOMAC cells (MOI of 1:100, high cell density of 10^6 cells/mL).

A.

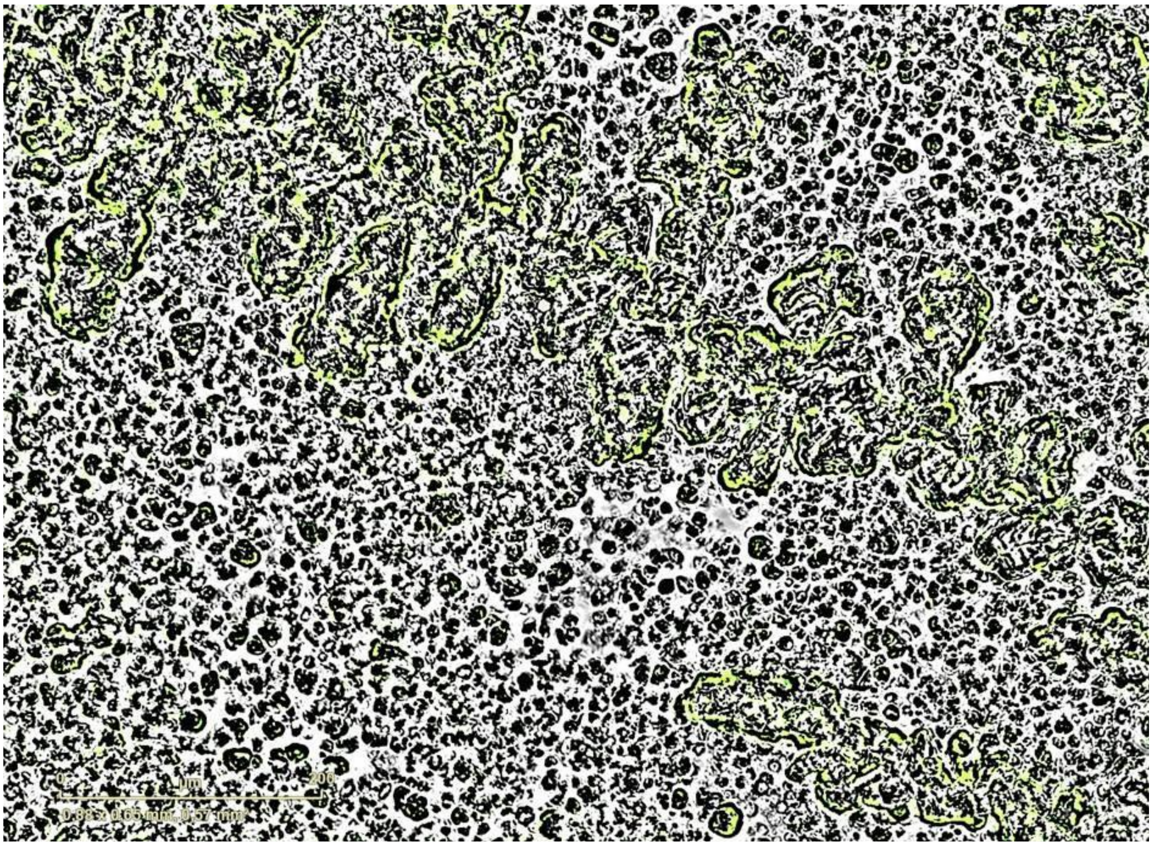

B.

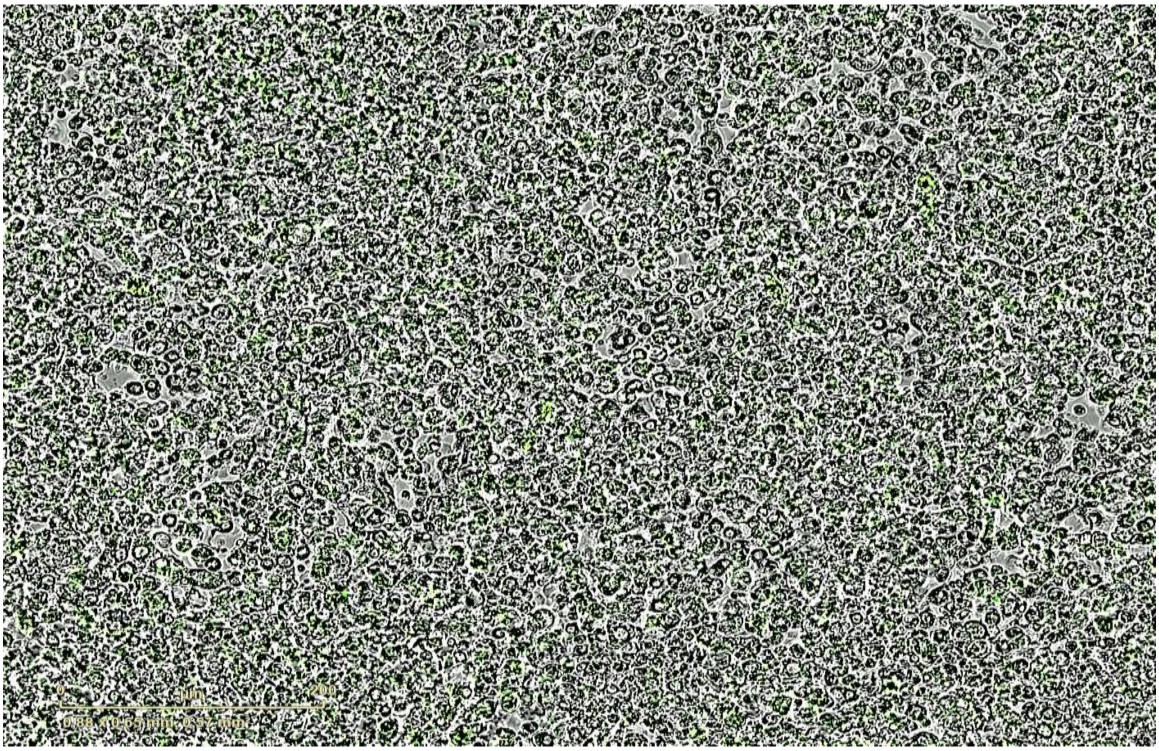

Supplement: Supplementary file 1 [file ijms-27-09640-s001.zip › ijms-4613167-supplementary.pdf]
